# Volatile, Microbial, and Sensory Profiles and Consumer Acceptance of Coffee Cascara Kombuchas

**DOI:** 10.3390/foods12142710

**Published:** 2023-07-15

**Authors:** Amanda Luísa Sales, Sara C. Cunha, Jéssika Morgado, Adriano Cruz, Thiago F. Santos, Isabel M.P.L.V.O. Ferreira, José O. Fernandes, Marco Antonio L. Miguel, Adriana Farah

**Affiliations:** 1 Núcleo de Pesquisa em Café Prof. Luiz Carlos Trugo (NUPECAFÉ), Laboratório de Química e Bioatividade de Alimentos, Instituto de Nutrição, Universidade Federal do Rio de Janeiro, Avenida Carlos Chagas Filho, 373, CCS, Bl. J, Rio de Janeiro 21941-902, Brazil; amandaalsalees@gmail.com (A.L.S.); jessikarmorgado@gmail.com (J.M.); thfsctaa@gmail.com (T.F.S.); 2 Laboratório de Microbiologia de Alimentos, Instituto de Microbiologia Paulo de Góes, Universidade Federal do Rio de Janeiro, Avenida Carlos Chagas Filho, 373, CCS, Bl. I, Rio de Janeiro 21941-902, Brazil; 3 LAQV-REQUIMTE, Laboratório de Bromatologia e Hidrologia, Departamento de Ciências Químicas, Faculdade de Farmácia da Universidade do Porto, 4099-030 Porto, Portugal; sara.cunha@ff.up.pt (S.C.C.); josefer@ff.up.pt (J.O.F.); 4 Instituto Federal de Educação, Ciência e Tecnologia do Rio de Janeiro, Rio de Janeiro 20260-100, Brazil; adriano.cruz@ifrj.edu.br

**Keywords:** coffee by-products, volatilome, coffee husk, black tea, fermentation, fermented beverages, novel foods

## Abstract

Given the substantial world coffee production, tons of coffee fruit cascara rich in bioactive compounds are discarded annually. Using this by-product to produce potentially healthy and acceptable foods is a sustainable practice that aggregates value to coffee production and may help improve people’s lives. This study aimed to elaborate kombuchas from coffee cascara tea, evaluate their microbial profile, and monitor the changes in the volatile profile during fermentation, together with sensory attributes and acceptance by consumers from Rio de Janeiro (*n* = 113). Arabica coffee cascaras from Brazil and Nicaragua were used to make infusions, to which black tea kombucha, a Symbiotic Culture of Bacteria and Yeasts (SCOBY), and sucrose were added. Fermentation of plain black tea kombucha was also monitored for comparison. The volatile profile was analyzed after 0, 3, 6, and 9 days of fermentation via headspace solid phase microextraction GC-MS. A total of 81 compounds were identified considering all beverages, 59 in coffee cascara kombuchas and 59 in the black tea kombucha, with 37 common compounds for both. An increase mainly in acids and esters occurred during fermentation. Despite the similarity to black tea kombucha, some aldehydes, esters, alcohols, and ketones in coffee cascara kombucha were not identified in black tea kombucha. Potential impact compounds in CC were linalool, decanal, nonanal, octanal, dodecanal, ethanol, 2-ethylhexanol, ethyl acetate, ethyl butyrate, ethyl acetate, β-damascenone, γ-nonalactone, linalool oxide, phenylethyl alcohol, geranyl acetone, phenylacetaldehyde, isoamyl alcohol, acetic acid, octanoic acid, isovaleric acid, ethyl isobutyrate, ethyl hexanoate, and limonene. The mean acceptance scores for cascara kombuchas varied between 5.7 ± 0.53 and 7.4 ± 0.53 on a nine-point hedonic scale, with coffee cascara from three-day Nicaragua kombucha showing the highest score, associated with sweetness and berry, honey, woody, and herbal aromas and flavors. The present results indicate that coffee cascara is a promising by-product for elaboration of fermented beverages, exhibiting exotic and singular fingerprinting that can be explored for applications in the food industry.

## 1. Introduction

Coffee is among the most consumed foods globally. In 2021/2022, the coffee world’s production was approximately 10 million tons [1]. Several steps are involved in coffee production. After the fruits are harvested, they may undergo different types of processing to release the seeds that are traditionally roasted and ground for coffee beverage extraction. While in wet processing the skin and pulp are fermented or enzymatically digested to release the seeds, in dry and semi-dry processing, they are mechanically separated from the seeds after washing and drying [2]. On a dry weight basis, coffee pulp alone corresponds to approximately 26–30% of coffee fruit, the skin approximately 10–12%, and the seeds 50–55% [3]. Therefore, a significant amount of biomass, which has been mainly considered “waste” material by the coffee industry, is currently discarded without further valorization [4].

Coffee cascara is rich in bioactive compounds such as chlorogenic acids, anthocyanins, and other flavonoids [5]. In Europe, coffee cascara was considered a novel food from 2005 until recently, when it received authorization by the European Food Safety Authority (EFSA) to be used as a food in the European market [6]. Since then, it has been incorporated in bread production as flour [7], added to food matrices like yogurts [8], or used for infusion preparation [2,9]. In the USA, it has been marketed as natural cascara tea.

Fermented foods are defined as foods made via desired microbial growth and enzymatic conversions of food components. Microorganisms determine the course and outcome of fermentation processes and contribute to the development of the characteristic properties of the final fermented food [10]. Kombucha is a fermented beverage traditionally consumed in Asia for centuries, and, as trade routes expanded, it reached Europe [11] and other Western countries. It is mainly made of sweetened green and/or black *Camellia sinensis* tea and a symbiotic culture (SCOBY), which comprises mainly acetic acid bacteria and yeasts, in addition to minor microorganisms [12].

Several potential benefits of traditional kombucha have been reported in vitro and in animal studies, such as antibacterial activity [13,14]; anticarcinogenic activity for colon breast, lung, and prostate cancers [13,14,15,16]; and a hypoglicemiant effect and weight loss in diabetic rats [17]. Most of these effects are related to the relevant antioxidant [18,19,20] and anti-inflammatory activities [15,20] inherent to *C. sinensis*. But kombucha brings about the benefits of being a cold beverage that can be consumed at any time in warm weather, replacing cold drinks. It also increases bioactive compounds’ bio-accessibility [21]. In recent years, kombucha’s popularity has exploded globally along with the healthier foods trend. The global kombucha market size was valued at USD 2.64 billion in 2021 and is expected to expand at an annual growth rate (CAGR) of 15.6% from 2022 to 2030 [22].

Recently, new raw materials have been added to or have even replaced *C. sinensis* in the production of kombucha and kombucha-like products. This includes food industry by-products, as sustainable and healthy alternatives [23]. So far, there are no reports on coffee cascara kombucha, other than our recent publication on its potential bioactivity [20]. Considering the potential health-promoting qualities of coffee cascara and the prospect of sustainability in coffee production, this study aimed to elaborate kombuchas from coffee fruit cascara infusions with different fermentation intensities and characterize the SCOBY microbial profiles and changes in the beverages’ volatile profile during fermentation, associating these changes with their sensory attributes. We also evaluated the acceptance of the beverages by consumers in Rio de Janeiro.

## 2. Materials and Methods

### 2.1. Samples

The lead commercial *C. sinensis* black tea (BT) was purchased in a food market in Rio de Janeiro, Brazil; dry and wet processed *Coffea arabica* cascara (CC) teas were acquired directly from producers in Domingos Martins, in the Caparaó Mountains region of Espírito Santo, Brazil (CCB), and from Matagalpa, Nicaragua (CCN), respectively.

### 2.2. Kombucha Consortium, Black Tea, and Coffee Cascara Kombucha

The kombucha consortium was part of the collection of the Microbiology Institute of the Federal University of Rio de Janeiro, Brazil. Previously cultivated in green tea, the consortium was fermented three times in black tea or coffee cascara tea infusion before experimental use to stabilize the microbial consortium in these matrixes [24]. All kombucha beverages were prepared according to the classical kombucha protocol described by Nummer [25].

*Infusions*—Black tea (BT) and coffee cascara (CC) infusions were prepared at 3% (weight/volume—*w*/*v*), pouring water at 95 °C over the raw material and letting it steep for 10 min.

*Black tea kombucha*—Black tea kombucha (BT K) was prepared by mixing 10% (*v*/*v*) of black tea infusion, 10% (*w*/*v*) sugar (sucrose), and 2.5% (*v*/*v*) of the SCOBY. The mixture was allowed to ferment at 23 °C. Samples were collected before fermentation (day 0) and after 3, 6, and 9 days of fermentation. An extra sample with pH 2.8 ± 0.05 was collected after 14 days of fermentation to be used as a starter for the coffee cascara kombucha production.

*Coffee cascara kombucha*—Coffee cascara kombuchas (CC K) were prepared using 80% (*v*/*v*) of the coffee cascara infusion, 10% (*v*/*v*) of the black tea kombucha starter, 10% (*w*/*v*) sugar, and 2.5% (*w*/*v*) SCOBY. The mixture was allowed to ferment at 23 °C. Samples were collected before and after 3, 6, and 9 days of fermentation (d0, d3, d6, and d9, respectively).

### 2.3. pH, Total Titratable Acidity, Total Soluble Solids Determination, and Sugar Analysis

Triplicate samples were prepared for analysis. pH was measured using a pH meter (Kasvi K39-0014PA, São José dos Pinhais, Paraná, Brazil). Total titratable acidity was determined via titration with 0.1 N NaOH and phenolphtalein as an indicator, according to Adolfo Lutz Institute [26]. Results were expressed in mEq/L. Total soluble solids were evaluated using a handheld refractometer (Pocket Refractometer Pal–1, ATAGO, Tokyo, Japan). Results were expressed in °Brix.

Sucrose was analyzed using a High Performance Liquid Chromatography Refractive Index Detector (RID) system (mod.# 2414, Waters, Milford, MA, USA), according to Wischral et al. [27], using a Hi-Plex column H 8 μm (300 × 7.7 mm) (Agilent, Santa Clara, CA, USA) at 30 °C with 20 μL of injection volume and H_2_SO_4_ 0.005 mol/L as mobile phase at 0.4 mL/min. For glucose and fructose, the column temperature was 60 °C, and the mobile phase flow was 0.6 mL/min. Calibration curves of external standards were used for the identification and quantification of all sugars (Supplementary Material Figure S1).

### 2.4. Analyses of Volatile Organic Compounds

Extraction of the volatile organic compounds from the infusions and kombuchas was performed via headspace solid-phase microextraction (HS-SPME), using a 50/30 μm divinylbenzene/carboxen/polydimethylsiloxane fiber (DVB/CAR/PDMS, Supelco^®^). The analyses were performed in a gas chromatographer (Agilent, 6890 Little Falls, DE, USA) coupled to a mass spectrometer (Agilent 5975) (GC-MS) and equipped with an auto analyser Combi-PAL (CTC Analytics, Zwingen, Switzerland), according to the methodology described by Wang et al. [28]. Before use, the fiber was conditioned according to the manufacturer’s recommendations. Two milliliters of each sample were placed in a 20 mL SPME vial, which was immediately sealed with silicone septa and conditioned for 5 min at 50 °C under continuous agitation. Then, the fiber was exposed to the vial headspace for 30 min, in agitation at 400 rpm, and heated at 50 °C. After this period, the fiber was retracted and inserted into the chromatographic injector, in splitless mode, for 2 min, for desorption of volatile compounds, with the aid of a carrier gas (helium), and transferred directly into the chromatographic column (SPB-5, 60 m × 0.32 mm, film thickness df = 1 μm 5% diphenyl—95% dimethyl polysiloxane, Supelco, Bellefonte, PA, USA) at 1 mL min/ for 10 min, 250 °C. The chromatographic separation conditions were: 40 °C for 3 min, ramped to 200 °C at 5 °C/min, subsequently ramped to 250 °C at 10 °C/min, and held at final temperature for 3 min. The transfer line, ion source, and MS quadrupole temperatures were 250, 230, and 150 °C, respectively. Electron impact mass spectra were measured at the acceleration energy of 70 eV. Data acquisition was performed in full-scan mode from *m*/*z* 50 to 550. Analytes were tentatively identified using the linear retention indices (LRIs) and confirmed using the National Institute of Standards and Technology (NIST V2.2, Gaithersburg, MD, USA) library database [29]. Agilent Chem Station (Agilent Technologies, Santa Clara, CA, USA) was used for data collection and processing. The LRI of each compound was calculated using the respective retention time (RT) compared against the RTs of a series of standard n-alkanes. The compounds were identified based on their LRI, the mass spectra of the NIST library [29], or authentic standards measured under the same conditions. To identify the compounds, substances with a probability greater than 50% were selected. To improve the accuracy of compounds’ identification, only those substances that provided a match factor higher than 600 and a match factor versus reversed match factor ratio greater than 0.8 were selected for data processing [2,30]. LRIs available from previous publications were also used for comparison. Analyses were performed in triplicate samples.

### 2.5. DNA Extraction, Amplicon Sequencing Data Analysis, and Library Preparation

DNA was extracted from the liquid and the SCOBY samples after 14 days of fermentation (starter culture) for black tea kombucha, and after 9 days of fermentation for all other beverages, following the protocol developed by Yamanaka et al. [31] and described in detail by Sales et al. [20].

### 2.6. Sensory Analysis

The study was approved (approval # 4.513.606) by the Ethical Committee of Clementino Fraga Filho University Hospital at Federal University of Rio de Janeiro and fully explained to the subjects who gave their written informed consent before participation. One hundred and fourteen consumer assessors took part in the Acceptance, Purchasing Intention, and Rate All That Apply (RATA) tests. They were students, teachers, visitors, and employees at the Federal University of Rio de Janeiro-UFRJ Health Sciences Center. Eligible criteria were people who consumed kombucha or sparkling beverages, such as sparkling water, soft drinks, cider, and sparkling wine. Because part of the test was performed during the COVID-19 pandemic, we asked if the person had a positive diagnosis for the disease and, consequently, the loss of taste and/or smell. These subjects were excluded from the study, as well as any other subjects with a condition that could affect sensory evaluation.

Assessors performed the tests on individual benches in the UFRJ Food and Dietetics Lab under white light. Before receiving the samples, demographic information was collected, including gender, age, educational level, occupation, family monthly income, and frequency and habits of kombucha and sparkling beverage consumption. Approximately 30 mL of kombucha was presented at 4 °C in 40 mL acrylic cups with dimensions 43 × 56 mm, coded with three-digit random numbers, and distributed in a balanced way to avoid the consistent influence of neighboring samples on the sensory perception. Crackers and spring water at room temperature were offered between samples to clean the palate.

#### 2.6.1. Consumer Acceptance and Purchase Intention

Assessors evaluated the infusions using the nine-point hedonic scale ranging from 1 (extremely disliked) to 9 (extremely liked), followed by a five-point purchase intention scale ranging from 1 (certainly would not buy) to 5 (certainly would buy) [32]. The Acceptability Index (AI) was calculated using the following equation: AI = (X × 100)/N, where: X = average score given by assessors and N = highest score given by assessors. An AI equal to or greater than 70% was considered satisfactory [32].

#### 2.6.2. Rate All That Apply (RATA)

After marking the hedonic scales, assessors were given a pre-prepared checklist with 34 sensory attributes related to appearance, aroma, flavor, and mouthfeel, which were identified in a preliminary session performed by a trained panel. The sensory panel consisted of nine trained assessors (aged 28–58) with a minimum of 200 h of experience in evaluating different food products and 30 h of experience in evaluating fermented beverages and infusions. In order to generate sensory descriptors, six samples of kombucha (three from this study and three traditional ones) were presented to assessors. First, assessors were asked to generate their individual descriptors using a modified grid method [33]. Via open discussion with the panel leader, assessors agreed on the best descriptors to fully describe the samples, their definitions, and how to evaluate those [34]. They were organized according to alphabetical order as follows: for aroma, citric, fermented, floral, herbal, honey, raisin, red fruits, rosé wine, syrup, woody, and yellow fruits; for taste, acid/sour, bitter, and sweet; for flavor, alcoholic, apple vinegar, beer yeast, hibiscus, prune, and vinegar; and for mouthfeel, texture and aspect, astringent, brown color, clear, fizzy, full-bodied, opaque/matte, refreshing, sparkling, and watery. Assessors were required to select all terms they considered appropriate to describe the infusions. Considering that kombucha samples exhibited different intensities of aroma, taste, and flavor attributes, we investigated whether assessors perceived such differences by asking them to score the attributes according to their intensity (RATA scores: 1 = low intensity; 2 = medium intensity; and 3 = high intensity).

### 2.7. Statistics

Analysis of variance (ANOVA), followed by Tukey’s test, was used to compare physicochemical analysis and acceptance and purchase intention tests results. Differences were considered significant when *p* ≤ 0.05 (Version 8.4.2, Informer Technologies, Los Angeles, CA, USA).

RATA scores were treated by ANOVA, using the arithmetic mean values of the sensory descriptors for all assessors. Non-applicable attributes were marked as intensity 0 [35,36]. Cluster analysis based on the hierarchical grouping of acceptance scores was carried out to identify segments of consumers with similar likings [2].

Principal Component Analysis (PCA) was performed to understand the evolution of volatile compounds identified as relevant for RATA attributes on CCB and CCN samples. For this purpose, the free software R (R version 4.2.2, R Studio team, 2022) was used.

## 3. Results and Discussion

### 3.1. Physicochemical Parameters

The pH, total acidity, soluble solids, and sucrose content values in all kombucha beverages are presented in Table 1. In BT K, total acidity increased until day 9 (0.3 mEq/L), when pH was 3.4. pH and titratable acidity values are within the range observed in the literature [14,37]. The increase in acidity is caused by the fermentation process, when bacteria and yeasts produce invertase that metabolizes sucrose into several organic acids, mainly acetic, glucuronic, lactic, and citric acids, and the monosaccharides glucose and fructose [38,39].

Considering the CC K, while in CCB K acidity remained stable (0.1 mEq/L), in CCN K acidity increased slightly during fermentation (Table 1). Nevertheless, in both CC Ks, no significant differences were observed in pH from d3 to d9. The range of pH values in these kombuchas is considered safe for human consumption [25]. Values below pH 2.5 indicate hazardous concentrations of acetic acid; likewise, values higher than 4.2 may affect the beverage’s microbiological safety [25].

Results are in accordance with previous studies using novel ingredients to prepare kombucha beverages [40] and agree with the fact that kombucha’s pH tends to stabilize after a few fermentation days, given the buffer effect caused by the organic acids and carbon dioxide formed in the fermentation process [38,40]. According to Ulusoy and Tamer [40], the obtained aqueous solution of carbon dioxide dissociates and produces the amphiprotic hydrocarbonate anion (HCO_3_^−^), which easily reacts with hydrogen ions (H+) from organic acids, preventing further changes in the (H^+^) concentration and contributing to a buffer character in the system.

Fermentation caused 20% decrease, on average, in the content of sucrose during fermentation (Table 1), with a small increase in glucose (0.84–1.09 g/100 mL) and fructose (0.3–0.5 g/100 mL) concentrations. As expected, a decrease in soluble solids also occurred, accompanying the decrease in sucrose concentration in the culture media over time [41].

### 3.2. Microbial Taxonomy

**Figure 1:**
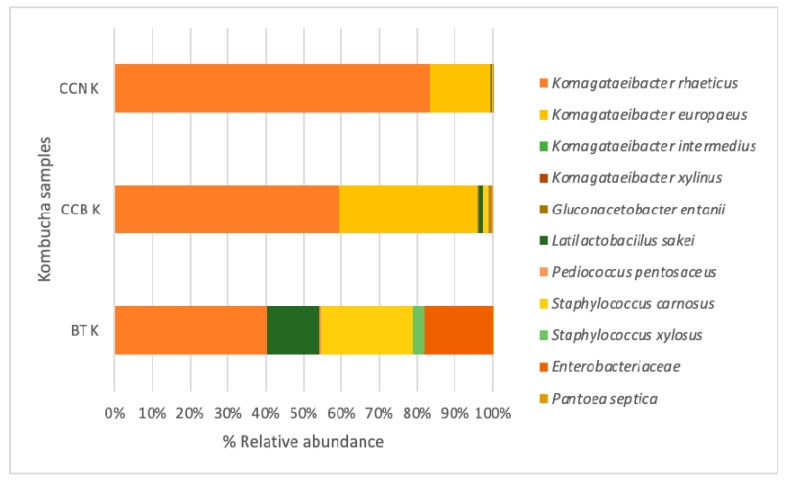
Bacterial composition of the solid and liquid phases of black tea and coffee cascara tea kombuchas’ consortia after 14 days (starter) and 9 days of fermentation, respectively. Note: BT K—black tea kombucha; CCB K—coffee cascara kombucha from Brazil; CCN K—coffee cascara kombucha from Nicaragua.

This analysis was performed to characterize the consortium and allow reproducibility, considering that the SCOBY composition may differ worldwide. Although all SCOBYs contain mainly acetic acid bacteria and yeasts, the profile of minor components may change according to the food matrices, and microorganisms may contribute differently to the changes in the chemical composition and physiological effects of kombucha [24,42]. The microbial community of the starter culture (BT K d14) and the final liquid and biofilm composition of both CCKs d9 were characterized (Figure 1). Data analysis of the 16S rRNA gene sequence revealed two bacterial phyla in all samples, Proteobacteria and Firmicutes. Proteobacteria was the most abundant phyla, especially in CCKs, with a percentage higher than 90%, in agreement with previous black tea kombuchas [43,44,45].

In all kombuchas, *Komagataeibacter*—an acetic acid bacteria and the most efficient bacterial cellulose producer [46]—was the most abundant genus observed in the liquid and biofilm, which is also in accordance with previous studies characterizing kombucha cultures [43]. In the starter culture, only the species *Komagataeibacter rhaeticus* was identified, with about 40% of the total number of bacteria. This is known to be one of the most abundant bacterial members among the kombucha fermenting agents [24,47]. The *Komagateibacter* genus has been positively correlated to the presence of ketones and aldehydes in a type of vinegar [48]. In wine, it was associated with cream, green grass, jams, and tropical fruit attributes [49].

In CC Ks, *K. rhaeticus* comprised more than 80% and 60%, respectively, of CCB and CCN K microorganisms contained in the liquid and solid cultures. *K. rhaeticus*, *K. europaeus* (15–36%), *K. intermedius* (0.2%), *K. europaeus,* and *K. intermedius* have previously been identified in black tea kombucha [24,50,51]. *Gluconacetobacter entanii* (0.5%) was identified in all CC Ks. The *Gluconacetobacter* genus has been previously detected in BT Ks [24,45], but it can also be isolated from other fermented food matrixes [52] including *G. entanii* in rooibos tea kombucha [53]. Also, this genus has useful characteristics to be combined with yeast strains for glucuronic acid production [54].

In the present study, *Staphylococcus* (24%), Enterobacteriaceae (18%), *Latilactobacillus* (15%), and *Pediococcus* (0.4%) were observed in BT K and not in CC K, showing that these microorganisms were in the BT leaves. The family Enterobacteriaceae comprises a very large group of morphologically and physiologically similar bacteria, such as *Escherichia coli* and *Salmonella typhimurium*. They are of great importance; while some of these organisms are involved in food spoilage, others are food-borne pathogens, and some are indicators of fecal contamination of food products [55]. Because near-boiling water is used for infusion preparation, we can consider that the beverages were microbiologically safe in relation to Enterobacteria contamination. In the study by DePaula et al. [2], the infusion of contaminated coffee cascara tea presented zero count of viable thermo-tolerant microorganisms. These bacteria are heat sensitive and are not viable at temperatures above 45 °C. An additional Enterobacteriaceae strain identified in the starter and in CCB K was *Pantoea septica* [56]. The *Pantoea* spp. genus was previously identified in grape cultivar for wine production and was positively correlated with straight-chain fatty alcohols, aromatic aldehydes, and terpenes in wine [49].

Low percentages of *Latilactobacillus* and *Pediococcus* (0.08–0.9%), Enterobacteriaceae (0.06–0.6%), and *Staphylococcus* (1.3%) were observed in CC Ks. Two lactic acid bacteria were identified in BT K and CCB K, *Latilactobacillus sakei* and *Pediococcus pentosaceus.* In a model kimchi, *L. sakei* produced volatile compounds such as hexanal, acetic acid, and geranyl acetone [57]. *Pediococcus pentosaceus* has been used to ferment tilapia surimi, and some of the main volatile compounds were the aldehydes hexanal, nonanal, heptanal, octanal, decanal, undecanal, and benzaldehyde [58]. *Staphylococcus carnosus* and *Staphylococcus xylosus* were identified in BT K and CCKs. They are coagulase-negative *Staphylococcus* spp. strains commonly found in diversified fermented food products as an integral part of the natural flora and are often recognized as non-infective microbiota. They can also attribute acidic and buttery tastes to fermented meats [59].

Regarding yeasts, their metabolism is not only responsible for the production of ethanol but also for the formation of several hundreds of flavor-active compounds, imparting their characteristic aroma and flavor to fermented beverages. The production and concentration of metabolites, desirable or not (off-flavors), formed during fermentation depends on the contribution of particular yeast species or strains. Thus, yeast communities have great potential to shape the aroma and flavor of fermented beverages [60].

**Figure 2:**
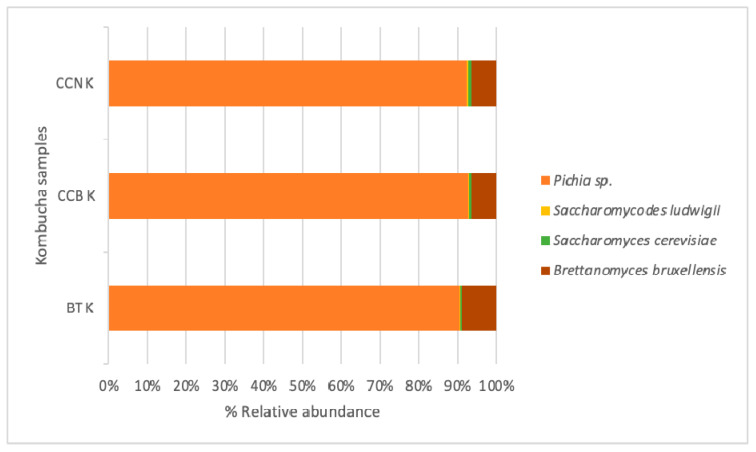
Yeast composition of the solid and liquid phases of the black tea and coffee cascara kombuchas’ consortia after 14 days (starter) and 9 days of fermentation, respectively. Note: BT K—black tea kombucha; CCB K—Brazil coffee cascara kombucha; CCN K—Nicaragua coffee cascara kombucha.

ITS1 analysis indicated that the most abundant phyla were Ascomycota (Figure 2). *Pichia* was the predominant yeast genera with an abundance higher than 70%, followed by *Saccharomyces* (>2%). The *Brettanomyces bruxellensis* strain (5%) was present in all kombuchas. Other non-saccharomyces strains comprised 0.4% of total yeasts. They were present in all fermented beverages.

*Pichia* sp. is one of the main yeast genera found in kombuchas [43,45,51]. *Pichia* species are generally applied in wine making to improve aroma composition [61]. The main *Pichia* strains identified in BT and CC kombuchas were *Pichia fermentans* and *Pichia kluyveri.*

Although in this study *Brettanomyces bruxellensis* was not as abundant as Pichia sp., it is the most common yeast identified in kombucha tea and SCOBY [24,47,62]. Brettanomyces yeasts can strongly affect the aroma of fermentation products. Many different positive and negative attributes, including apple, floral, tropical fruit, citrus and/or spicy, cracker biscuit, clove, mousy, barnyard, smoky, plastic, phenolic, medical, “band-aid”, metallic, humid leather, sweaty, and goat-like are used to describe the (often pungent) aroma profile of these strains [63]. In kombucha, *Brettanomyces bruxelensis* can produce high amounts of alcohols and acids, such as isoamyl alcohol, phenylehtyl alcohol, isovaleric acid, hexanoic acid, octanoic acid, and lauric acid [64].

*Saccharomyces* sp. is the major yeast genus involved in the production of alcoholic beverages [65]. *Saccharomyces* sp. strains have been previously identified in the liquid and pellicle of kombuchas [43,45,51]. During fermentation, *Saccharomyces cerevisiae* (0.4–1% in our kombuchas) produces a broad range of aroma-active substances that are vital for the complex flavor of fermented beverages, with esters being the most important compounds with industrial purposes, since they are responsible for fruity, candy, and perfume-like aromas of alcoholic fermented beverages [66]. *Saccharomycodes ludgwigii* is considered detrimental to the winemaking process and contaminates ciders because it can produce in fermented beverages the volatiles ethyl acetate, isoamyl acetate, and acetaldehyde, which may confer negative undertones to wine when exceeding their respective thresholds of perception [67,68].

It is worth noting that some bacteria and genera identified in CC K in this study (*Acetobacter, Pediococcus pentosaceus*, Enterobacteria, and *Saccharomyces* sp.) are common to coffee fruit and seeds since coffee cascara is a postharvest by-product [69,70]. However, as mentioned previously, these microorganisms were probably not viable for taking part in the fermentation process given that coffee cascara was subjected to near-boiling temperatures during the infusion preparation.

### 3.3. Volatile Organic Compounds

**Figure 3:**
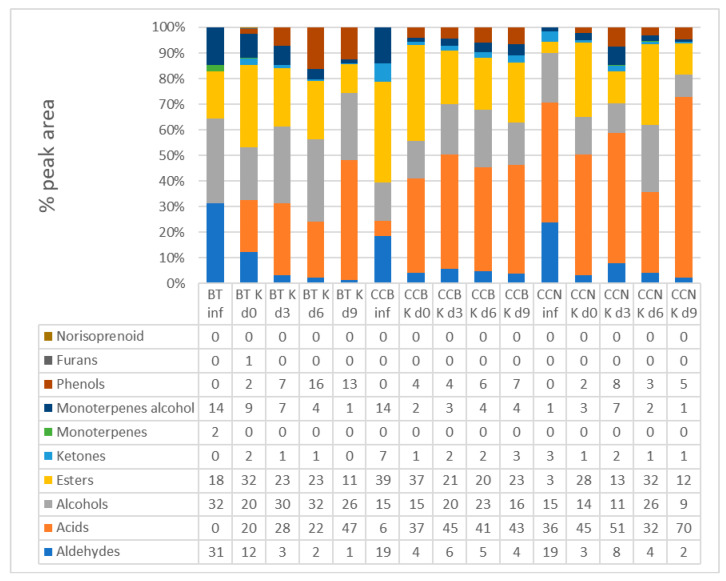
Changes in the relative peak areas (%) of volatile organic compounds in black tea and coffee cascara infusion and kombucha beverages, grouped into chemical classes. Note: BT: black tea; Inf: infusion; CCB: coffee cascara from Brazil; CCN: coffee cascara from Nicaragua.

Figure 3 compares the relative peak areas of organic volatile compounds (grouped into classes) in infusions and kombuchas made with black tea and coffee cascara samples. Total ion chromatograms are available as Supplementary Material (Figure S2).

Aldehydes represented 1–12% of total peak areas in BT K and 2–6% in coffee cascara kombuchas, decreasing as fermentation progressed in all samples, given the transformation into the corresponding derived alcohols [71]. Alcohols represented 26–58% of the total peak area in the BT K and 10–58% in CC Ks, decreasing up to d9. The largest peak areas were observed in CCB K (58%, 45%, 50%, and 20% at d0, d3, d6, and d9, respectively). The amount of alcohol at d0 of fermentation in both Ks is derived from BT and CC raw material and the starter [72,73]. Acids showed the largest peak areas (21–46% of the total peak area in the BT K and 31–70% in coffee cascara kombuchas), with a higher percentage in CCN K d9 (70%). Volatile acids are produced during alcoholic and acetic fermentation, via the symbiosis between acetic acid bacteria and yeasts in SCOBY [72]. Esters represented 12–33% of the total peak areas in the BT K and 9–38% in CC Ks. In all CC Ks, an increase in the number of esters was observed during fermentation, although the area of this chemical class decreased from d0 to d9.

Ketones comprised 0.3–2% of total peak areas in the BT K and 0.1–3% in CC Ks, monoterpenes represented 0–1% in the BT K and 0–0.5% in CC Ks, and monoterpene alcohols 1–12% in the BT K and 0.8–7% CC Ks.

#### 3.3.1. Black Tea Kombucha

The volatile compounds identified in the infusions and kombuchas are presented in Table 2. The total chromatogram peak areas can be observed in Figure 3.

The importance of aldehydes, alcohols, and esters for black tea aroma has been reported [73]. In the present study, the following volatile compounds identified as key odorant compounds, according to their aroma activity values (OAV), were identified in the BT infusion: benzaldehyde, linalool, phenylethyl alcohol, hexanal, nonanal [74], and benzaldehyde. Other common compounds in black tea identified in the infusions and the BT K d0 were dihydroactinidiolide and theaspirane [73]. Theaspirane and the furan dihydroactinidiolide are carotenoid-derived aroma compounds in black tea [74].

**Table 2:** Volatile compounds identified in black tea kombucha.

Volatile Compound	Odor Description	CAS ^a^	ELRI ^b^	LRI ^c^	BT	BT K d0	BT K d3	BT K d6	BT K d9
*Aldehydes*									
2-Hexenal	Apple, green, sweet, almond, fruity, leafy, plum, vegetable ^1,2^	505-57-7	914	939	□	■	□	□	□
2-Methylbutyraldehyde	Musty, cocoa, phenolic, coffee, nutty, malty, fermented, fatty, alcoholic ²	96-17-3	924	927	■	■	□	□	□
Benzaldehyde *	Almond, burnt sugar, tropical fruit ^1,2^	100-52-7	904	914	□	■	□	□	□
Dodecanal	Soapy, waxy, aldehydic, citrus, green, floral ^2^	112-54-9	927	958	■	■	■	■	■
Ethanal/Acetaldehyde	Pungent, ether, fresh, fruity, musty ^1,2^	75-07-0	956	983	□	■	■	■	■
Heptanal	Fat, citrus, rancid, fresh, aldehydic, green, herbal, wine-lee, ozone ^1,2^	111-71-7	784	864	□	□	□	□	■
Hexanal *	Grass, tallow, fat, fresh, green, aldehydic, leafy, fruity, sweaty ^1,2^	66-25-1	920	943	■	■	■	□	□
Isobutyraldehyde	Pungent, malt, green, fresh, aldehydic, floral, green ^1,2^	78-84-2	910	916	□	■	□	□	□
Isovaleraldehyde	Ethereal, aldehydic, chocolate, peach, fatty ^2^	590-86-3	910	912	□	■	■	□	□
Nonanal *	Fat, citrus, fresh, orange, green ^1,2^	124-19-6	875	897	■	■	■	■	■
Octanal	Citrus, soap, lemon, herbal, green, honey ^1,2^	124-13-0	856	927	■	■	■	■	■
2-Phenylethanal	Honey, floral, rose, sweet, powdery, fermented, chocolate, earthy, hawthorn, green, hyacinth, clover, cocoa ^1,^	122-78-1	925	942	■	■	■	■	□
Tetradecanal	Fatty, waxy, amber, incense, dry, citrus, peel, musk ^2^	124-25-4	705	826	□	□	□	■	■
*Acids*									
2-methylbutanoic acid	Pungent, acid, Roquefort cheese ^2^	116-53-0	806	832	□	□	□	■	■
Acetic acid	Acidic, sour, pungent, vinegar ^1,2^	64-19-7	943	956	□	■	■	■	■
Caproic acid	Sweat, sour, fatty, cheese ^1,2^	142-62-1	884	906	□	□	■	■	■
Decanoic acid	Rancid, fat, unpleasant, rancid, sour, fatty, citrus ^1,2^	334-48-5	907	914	□	■	■	□	■
Isovaleric acid	Sweat, acid, rancid, sour, stinky, feet, cheese, tropical ^1,2^	503-74-2	876	887	□	□	■	■	■
Lauric acid	Metal, mild, fatty, coconut, bay, oil ^1,2^	143-07-7	755	841	□	□	□	□	■
Nonanoic acid	Green, fat, waxy, dirty, cheese, cultured, dairy ^1,2^	112-05-0	892	898	□	■	■	■	■
Octanoic acid	Acid, sweat, cheese, fruit notes ^1,2^	124-07-2	917	922	□	■	■	■	■
*Alcohols*									
1-penten-3-ol	Ethereal, horseradish, green, radish, chrysanthemum, vegetable, tropical, fruity ^2^	616-25-1	737	869	□	■	□	□	□
2-Ethylhexanol	Rose, green, citrus, fresh, floral, oily, sweet ^1,2^	104-76-7	954	954	■	■	■	□	■
(S)-(–)-2-methyl-1-butanol	Ethereal, fresh ^2^	1565-80-6	807	858	□	□	□	□	■
2-methyl-1-butanol	Malt, wine, onion, ethereal, fusel, alcoholic, fatty, greasy, whiskey, leathery, cocoa ^1,2^	137-32-6	918	923	□	■	■	■	□
3-methyl-1-butanol/isoamyl alcohol	Whiskey, malt, burnt, fusel, oil, alcoholic, fruity, banana ^1,2^	123-51-3	903	909	□	□	□	■	■
Phenylethyl alcohol *	Honey, spice, rose, lilac, floral, fresh ^1,2^	60-12-8	917	939	□	■	■	■	■
(E)-2-Hexenol	Green, leaf, walnut, fresh, fruity, unripe, banana ^1,2^	928-95-0	774	799	□	■	□	□	□
Amyl alcohol	Fusel, oil, sweet, balsam ^2^	71-41-0	811	870	□	□	■	□	□
Cedrol	Cedarwood, woody, dry, sweet, soft ^2^	77-53-2	611	753	■	□	■	□	□
Ethanol	Sweet ^1^	64-17-5	973	973	□	■	■	■	■
Octanol	Moss, nut, mushroom, waxy, green, orange, aldehydic, rose ^1,2^	111-87-5	810	870	□	□	□	■	□
*Esters*									
2-methylbutyl acetate	Fruit, over-ripe, sweet, banana, juicy ^1,2^	624-41-9	845	899	□	□	□	□	■
Ethyl 2–methylbutyrate	Sharp, sweet, green, apple, fruity ^2^	7452-79-1	772	871	□	□	□	□	■
Ethyl acetate	Pineapple, ethereal, fruity, sweet, weedy, green ^1,2^	141-78-6	951	961	□	■	■	■	■
Ethyl decanoate	Grape, sweet, waxy, fruity, apple, oily, brandy ^1,2^	110-38-3	947	983	□	□	■	■	■
Ethyl isobutyrate	Sweet, rubber, ethereal, fruity, alcoholic, fusel, rummy ^1,2^	97-62-1	754	786	□	□	□	□	■
Ethyl laurate	Leaf, sweet, waxy, floral, soapy, clean ^1,2^	106-33-2	876	901	■	□	□	■	■
Ethyl miristate	Sweet, waxy, violet, orris ^2^	124-06-1	815	848	■	□	□	■	□
Ethyl octanoate	Fruit, banana, pear ^1,^	106-32-1	873	930	□	□	□	■	■
Ethyl phenylacetate	Fruit, sweet, floral, honey, rose, balsam, cocoa ^1,2^	101-97-3	840	870	□	□	□	■	■
Isopropyl myristate	Faint, oily, fatty ^2^	110-27-0	681	767	■	■	□	□	□
Isopropyl palmitate	Fat, bland, oily ^1,2^	142-91-6	780	735	□	□	■	■	□
Methyl salicylate	Peppermint ^1^	119-36-8	897	958	■	■	■	■	□
*Ketones*									
3,5-Octadienone	Fruity, fatty, mushroom ^2^	38284-27-4	680	835	■	■	□	□	□
Acetylpropionyl	Pungent, sweet, butter, creamy, caramel, nutty, cheese ^2^	600-14-6	834	912	□	□	■	□	□
Geranyl acetone	Magnolia, green, fresh, fruity, waxy, rose, woody, tropical ^1,2^	3796-70-1	648	802	□	■	□	□	□
β-damascenone *	Apple, rose, honey, tobacco, sweet ^1,2^	23726-93-4	832	927	■	■	■	■	■
*Monoterpenes*									
(Z)-Sabinene hydrate	Balsam ^2^	15537-55-0	818	828	■	■	□	□	□
γ-Terpinene	Terpineol, lilac ^2^	586-81-2	871	884	■	■	□	□	□
β-Ionone *	Seaweed, violet, flower, raspberry, woody, sweet, fruity, berry, tropical, beeswax ^1,2^	14901-07-6	815	825	■	■	■	■	■
*Monoterpenes alcohols*									
α-Terpineol	Oil, anise, mint, lemon, citrus ^1,2^	98-55-5	801	820	□	■	■	■	□
1-Terpinen-4-ol	Turpentine, nutmeg, must, pepper, woody, earth, musty, sweet ^1,2^	562-74-3	803	868	□	■	■	□	□
Linalool *	Citrus, flower, lavender, sweet, green ^1,2^	78-70-6	921	925	■	■	■	■	■
Linalool oxide	Flower, wood, musty, camphor, fenchyl, alcohol ^1,2^	60047-17-8	730	778	■	■	■	■	■
*Furans*									
Dihydroactinidiolide	Musk, coumarin ^2^	17092-92-1	772	851	■	■	■	■	■
Furfuryl alcohol	Burnt, alcoholic, chemical, musty, sweet, caramel, bread, coffee	98-00-0	806	849	□	□	□	□	■
*Pyrrol*									
1-Ethyl-1H-pyrrole-2-carboxaldehyde	Burnt, roasted, smoky ^2^	2167-14-8	792	832	■	■	□	□	□
*Phenols*									
4-Ethylguaiacol	Spice, clove, smoky, bacon, phenolic/medicinal ^1,2^	2785-89-9	898	918	□	■	■	■	■
4-Ethylphenol	Phenolic/medicinal, castoreum, smoke, guaiacol ^2^	123-07-9	917	922	□	■	■	■	■
*Norisoprenoid*									
Theaspirane	Tea, herbal, green, wet, tobacco, leaf, metallic, woody, spicy ^2^	36431-72-8	805	913	■	■	□	□	□

Note: BT: black tea; BT K: black tea kombucha; ^a^ CAS (Chemical Abstracts Service) Registry Number, available in the NIST database; ^b^ ELRI: Experimental Linear Retention Index; ^c^ LRI: Linear Retention Index based on literature and NIST database [29]; 100% of the compounds in the chromatogram were identified. Alkanes were excluded. * Impact compounds according to Kang et al. [73]. ^1^ http://www.flavornet.org (accessed on 7 June 2023) [75]; ^2^ http://www.thegoodscentscompany.com (accessed on 7 June 2023) [76]; d0, d3, d6, d9: days 0, 3, 6, and 9 of fermentation; ■ compound identified in the sample; □ not identified.

In BT K, the number of compounds increased from 22 to 59 from d0 to d9. During fermentation, microorganisms consume carbon sources, mainly sugar and similar molecules, to produce acids, alcohol, and other volatiles [77]. In BT K d0, only 4 acids were identified (acetic acid, decanoic acid, nonanoic acid, and octanoic acid). As fermentation continued, the number of acids, alcohols, and esters in the chromatograms increased, although the areas of alcohol and esters decreased. Regarding aldehydes, the percentage and number of volatile compounds decreased as fermentation progressed till d9. Furans and pyrrols were identified in the infusions and kombuchas. These volatile compounds can be generated via the Maillard reaction during the tea manufacturing process [78].

High concentrations of caproic, octanoic, and lauric acids and the terpenoids α-terpineol were previously identified in traditional kombucha fermented by its native microbial consortia [64]. In the present study, the presence of the phenols 4-ethylguiacol and 4-ethylphenol in black tea kombucha is possibly derived from the action of *Brettanomyces* yeasts and other microorganisms on non-volatile polyphenols [79]. These phenols can impart undesirable odors to wine [80]. Phenylethyl ethanol and isovaleric acid were previously identified in black tea kombuchas [80,81,82], while acetic acid, decanoic acid, isoamyl alcohol, cedrol, ethanol, ethyl acetate, ethyl laurate, ethyl miristate, β-ionone, and α-terpineol were identified in raw pu-erh tea kombuchas [83].

#### 3.3.2. Coffee Cascara Kombuchas

The volatile compounds identified in coffee cascara infusions and kombuchas are presented in Table 3. Considering CCB and CCN infusions, 24 and 28 volatile compounds were identified, respectively. The main chemical groups in CC infusions were alcohols, esters, aldehydes, acids, and ketones, in accordance with DePaula et al. [2] and Pua et al. [84]. Benzaldehyde, decanal, and 2-ethylhexyl salicylate were only identified in coffee cascara infusions and not in the kombuchas.

Coffee cascara infusions contained key odorants such as ethyl octanoate, nonanal, linalool, benzaldehyde, methyl salicylate, β-damascenone, and γ-nonalactone, previously identified in dried fruits such as prunes or raisins [85,86] and in cascara infusions [2].

The volatile profiles (Table 3) were similar in both coffee cascara infusions and kombuchas. Considering the kombucha beverages elaborated using CCB and CCN, 59 volatile compounds were identified. To date, there are no similar studies using coffee cascara as raw material for kombucha production, and therefore only comparison with similar beverages is possible. As observed in BT K, a progressive increase in the number of acids, alcohols, and esters, and other minor classes of volatile compounds, was observed during fermentation, while the number and area % of aldehydes decreased.

Thirty-nine volatile compounds were common to the BT K and CC Ks (which contained 10% BT K): ten aldehydes (2-methylbutyraldehyde, isovaleraldehyde, benzaldehyde, dodecanal, acetaldehyde, heptanal, hexanal, nonanal, octanal, and tetradecanal);five acids (decanoic acid, isovaleric acid, lauric acid, nonanoic acid, and octanoic acid), six alcohols (2-ethylhexanol, 2-methyl-1-butanol, (S)-(–)-2-methyl-1-butanol, cedrol, ethanol, and octanol), four monoterpenes alcohols (1-terpin-4-ol, α-terpineol, linalool, and linalool oxide), nine esters (ethyl acetate, ethyl decanoate, ethyl isobutyrate, ethyl laurate, ethyl miristate, ethyl octanoate, ethyl phenylacetate, isopropyl palmitate, and methyl salicylate), and two ketones (geranyl acetone and β-damascenone). Most of them were probably from the BT K starter.

Terpineol, trans-linalool oxide, isovaleric acid, isoamyl acetate, and hexanoic acid have been associated with significant aroma contributions to black tea kombucha because of their considerable concentrations [87]. Trans-linalool oxide, linalool, phenylethyl alcohol, hexanal, nonanal, benzaldehyde, and β-ionone, identified in CC Ks, have been reported as aroma-impact compounds in black tea infusions and kombuchas [73]. As aforementioned, the presence of the phenols 4-ethylguiacol and 4-ethylphenol in the cascara kombuchas is probably derived from the fermentative process by yeasts from the genus *Brettanomyces* [79].

α-hexylcinnamaldehyde, 1-dodecanol, 1-heptanol, hexadecanol, isopulegol, (E)-linalool oxide, (Z)-linalool oxide, ethyl butyrate, ethyl hexanoate, isoamyl acetate, and γ-nonalactone were only identified in coffee cascara kombuchas and not in black tea kombuchas.

**Table 3:** Volatile organic compounds identified in coffee cascara kombuchas.

Volatile Compound	Odor Description	CAS# ^a^	ELRI ^b^	LRI ^c^	CCB	CCB K d0	CCB K d3	CCB K d6	CCB K d9	CCN	CCN K d0	CCN K d3	CCN K d6	CCN K d9
*Aldehydes*														
α-Hexylcinnamaldehyde	Fresh, floral, green, jasmine, herbal, waxy ^2^	101-86-0	871	908	□	□	□	□	■	□	□	□	□	□
2-methylbutyraldehyde	Musty, cocoa, phenolic, coffee, nutty, malty, fermented, fatty, alcoholic ^2^	96-17-3	644	754	□	■	□	□	□	□	■	□	□	□
Isovaleraldehyde	Ethereal, aldehydic, chocolate, peach, fatty ^2^	590-86-3	777	842	■	■	□	□	□	□	■	□	□	□
Benzaldehyde *	Almond, burnt sugar, tropical fruit ^1,2^	100-52-7	736	885	■	□	□	□	□	■	□	□	□	□
Decanal	Sweet, citrus, floral, soap, orange peel ^1,2^	112-31-2	806	846	■	■	□	□	□	□	□	□	□	□
Dodecanal	Soapy, waxy, aldehydic, citrus, green, floral ^2^	112-54-9	929	963	■	■	■	■	■	■	■	■	■	■
Ethanal/Acetaldehyde	Pungent, ether, fresh, fruity, musty ^1,2^	75-07-0	980	982	□	■	■	■	■	□	■	■	■	■
Heptanal	Fat, citrus, rancid, fresh, aldehydic, green, herbal, wine-lees, ozone ^1,2^	111-71-7	789	863	■	■	■	□	□	□	□	□	□	□
Hexanal	Grass, tallow, fat, fresh, green, aldehydic, leafy, fruity, sweaty ^1,2^	66-25-1	854	892	■	■	□	□	□	■	■	□	□	□
Nonanal *	Fat, citrus, fresh, orange, green ^1,2^	124-19-6	911	918	■	■	■	■	■	■	■	■	■	■
Octanal	Citrus, soap, lemon, herbal, green, honey ^1,2^	124-13-0	906	937	■	■	■	■	■	■	■	■	■	■
Palmitaldehyde	Cardboard ^1,2^	629-80-1	769	843	■	■	□	□	□	□	□	□	□	■
Phenylethanal/phenylacetaldehyde *	Honey, floral, rose, sweet, powdery, fermented, chocolate, earthy, hawthorn, green, hyacinth, clover, cocoa ^1,2^	122-78-1	859	930	□	□	■	■	□	□	□	□	□	□
Tetradecanal	Fatty, waxy, amber, incense, dry, citrus, peel, musk ^2^	124-25-4	723	852	□	□	□	□	□	■	■	■	■	□
Undecanal	Waxy, soapy, floral, aldehydic, citrus, green, fatty, fresh, laundry ^2^	112-44-7	723	865	□	□	□	□	□	■	■	□	□	□
*Acids*														
Acetic acid	Acidic, sour, pungent, vinegar ^1,2^	64-19-7	943	956	□	■	■	■	■	■	■	■	■	■
Caproic acid	Sweat, sour, fatty, cheese ^1,2^	142-62-1	864	898	□	□	□	□	■	□	□	□	■	■
Decanoic acid	Rancid, fat, unpleasant, rancid, sour, fatty, citrus ^1,2^	334-48-5	923	948	■	■	■	■	■	■	■	■	■	■
Isobutyric acid	Rancid, butter, cheese, acidic, sour ^1,2^	79-31-2	831	861	□	□	□	□	□	■	■	□	□	■
Isovaleric acid	Sweat, acid, rancid, sour, stinky, feet, cheese, tropical ^1,2^	503-74-2	851	868	□	□	□	■	■	□	□	□	■	■
Lauric acid	Metal, mild, fatty, coconut, bay, oil ^1,2^	143-07-7	689	826	□	□	□	□	■	□	□	□	□	□
Nonanoic acid	Green, fat, waxy, dirty, cheese, cultured, dairy ^1,2^	112-05-0	897	904	■	■	■	■	■	■	■	■	■	■
Octanoic acid	Acid, sweat, cheese, fruit notes ^1,2^	124-07-2	922	928	□	□	■	■	■	■	■	■	■	■
*Alcohols*														
1-dodecanol	Earthy, soapy, waxy, fatty, honey, coconut ^2^	112-53-8	796	924	■	■	□	□	■	□	□	□	□	□
1-Heptanol	Musty, leafy, violet, herbal, green, sweet, woody, peony ^2^	111-70-6	796	834	□	□	□	□	□	□	□	■	■	■
2-Ethylhexanol	Rose, green, citrus, fresh, floral, oily, sweet ^1,2^	104-76-7	906	953	■	■	■	■	■	■	■	■	■	■
2-Methyl-1-butanol	Malt, wine, onion, ethereal, fusel, alcoholic, fatty, greasy, whiskey, leathery, cocoa ^1,2^	137-32-6	858	904	□	■	■	■	□	□	■	■	□	□
(S)-(–)-2–methyl-1-butanol	Ethereal, fresh ^2^	1565-80-6	796	847	□	■	□	□	□	□	■	□	□	□
3-Methyl-1-butanol/isoamyl alcohol *	Whiskey, malt, burnt, fusel, oil, alcoholic, fruity, banana ^1,2^	123-51-3	892	899	□	■	□	■	■	□	■	□	□	■
2-Nonen-1-ol	Sweet, fatty, melon, cucumber, vegetable ^2^	22104-79-6	636	741	■	■	□	□	□	□	□	□	□	□
(E)-2-Decen-1-ol	Waxy, fresh, air, citrus, rose, rue ^2^	18409-18-2	792	796	□	□	□	□	□	□	□	□	□	■
(E)-2-Octen-1-ol	Green, citrus, vegetable, fatty ^2^	18409-17-1	602	663	□	□	■	■	■	□	□	□	□	□
Phenethyl alcohol	Honey, spice, rose, lilac, floral, fresh ^1,2^	60-12-8	825	885	■	■	■	■	■	□	■	■	■	□
Cedrol	Cedarwood, woody, dry, sweet, soft ^2^	77-53-2	653	784	□	■	□	□	□	□	■	■	□	□
Ethanol	Sweet ^2^	64-17-5	945	945	□	■	■	■	■	■	■	■	■	■
Hexadecanol	Flower, wax, clean, greasy, floral, oily ^1,2^	36653-82-4	932	951	□	□	□	□	■	■	□	□	□	□
Isopulegol	Minty, cooling, medicinal, woody ^2^	89-79-2	812	816	□	□	□	□	□	■	■	■	■	□
Octanol	Moss, nut, mushroom, waxy, green, orange, aldehydic, rose ^1,2^	111-87-5	712	815	□	□	□	■	■	□	□	□	□	□
*Esters*														
2-Ethylhexyl salicylate	Mild, orchid, sweet, balsam ^2^	118-60-5	863	961	■	■	□	□	□	■	■	□	□	□
Ethyl 2-methylbutyrate	Sharp, sweet, green, apple, fruity ^2^	7452-79-1	877	895	□	□	□	□	■	□	□	□	□	■
Ethyl Acetate	Pineapple, ethereal, fruity, sweet, weedy, green ^1,2^	141-78-6	940	950	□	■	■	■	■	□	■	■	■	■
Ethyl butyrate	Apple, fruity, juicy, fruit, pineapple, cognac ^1,2^	105-54-4	765	848	□	□	□	□	□	□	□	□	□	■
Ethyl decanoate	Grape, sweet, waxy, fruity, apple, oily, brandy ^1,2^	110-38-3	915	928	□	□	■	■	■	□	□	□	■	■
Ethyl hexanoate *	Apple peel, fruit, sweet, pineapple, waxy, green, banana ^1,2^	123-66-0	909	912	□	■	□	□	■	□	■	□	□	■
Ethyl isobutyrate *	Sweet, rubber, ethereal, fruity, alcoholic, fusel, rummy ^1,2^	97-62-1	882	895	□	□	□	□	■	□	□	□	□	□
Ethyl laurate	Leaf, sweet, waxy, floral, soapy, clean ^1,2^	106-33-2	814	841	□	□	□	■	■	□	□	□	■	■
Ethyl miristate	Sweet, waxy, violet, orris ^2^	124-06-1	775	780	□	□	□	□	□	□	□	□	□	■
Ethyl octanoate *	Fruit, banana, pear ^1,2^	106-32-1	913	939	□	□	■	■	■	□	□	□	■	■
Ethyl palmitate	Wax, fruity, creamy, milky, balsamic, greasy, oily ^1,2^	628-97-7	791	833	■	□	□	□	□	■	□	□	□	□
Ethyl phenylacetate	Fruit, sweet, floral, honey, rose, balsam, cocoa ^1,2^	101-97-3	874	894	□	□	□	□	■	□	□	□	□	■
Homomenthyl salicylate	Mild, menthol ^2^	118-56-9	792	910	■	■	□	□	□	□	□	□	□	□
Isoamyl acetate *	Banana, sweet, fruity, solvent ^1,2^	123-92-2	772	878	■	■	□	□	■	□	□	□	□	□
Isopropyl palmitate	Fat, bland, oily ^1,2^	142-91-6	790	882	■	■	□	□	■	□	□	□	□	□
Methyl dihydrojasmonate	Floral, oily, jasmine, green, lactonic, tropical, natural ^2^	24851-98-7	785	823	□	□	□	□	□	■	■	□	□	□
Methyl palmitate	Oily, waxy, fatty, orris ^2^	112-39-0	762	848	□	□	□	■	■	□	□	□	□	□
Methyl salicylate	Peppermint ^1^	119-36-8	895	920	■	■	■	■	■	■	■	■	■	□
*Ketones*														
Geranyl acetone	Magnolia, green, fresh, fruity, waxy, rose, woody, tropical ^1,2^	3796-70-1	682	777	□	□	□	□	□	■	■	□	□	■
β-Damascenone *	Apple, rose, honey, tobacco, sweet ^1,2^	23726-93-4	878	944	■	■	■	■	■	■	■	□	□	□
*Monoterpenes*														
Limonene	Lemon, orange, citrus, herbal, terpene, camphor ^1,2^	138-86-3	861	893	□	□	□	□	■	■	■	■	■	■
γ-Nonalactone *	Coconut, peach, creamy, waxy, sweet, buttery, oily ^1,2^	104-61-0	859	895	■	■	■	■	■	■	■	■	■	■
*Monoterpenes alcohols*														
α-Terpineol	Oil, anise, mint, lemon, citrus ^1,2^	98-55-5	871	900	□	□	■	■	■	□	■	■	□	□
1-Terpinen-4-ol	Turpentine, nutmeg, must, pepper, woody, earth, musty, sweet ^1,2^	562-74-3	681	804	■	■	□	□	□	■	■	■	□	□
Linalool *	Citrus, flower, lavender, sweet, green ^1,2^	78-70-6	919	925	■	■	■	■	■	■	■	■	■	■
Linalool oxide	Flower, wood, musty, camphor, alcohol ^1,2^	60047-17-8	891	900	■	■	■	□	□	□	□	□	■	■
cis-Linalol oxide	Flower ^1^	5989-33-3	838	852	□	□	□	■	■	□	□	□	□	□
trans-Linalool oxide	Flower ^1,2^	34995-77-2	863	878	□	□	□	□	□	■	■	■	□	□
*Furans*														
Furfural *	Bread, almond, sweet, woody ^1,2^	98-01-1	924	924	■	■	□	□	□	■	■	□	□	□
*Phenols*														
4-Ethylguaiacol	Spice, clove, smoky, bacon, phenolic/medicinal ^1,2^	2785-89-9	862	881	□	■	■	■	■	□	■	■	■	■
4-Ethylphenol	Phenolic/medicinal, castoreum, smoke, guaiacol ^2^	123-07-9	858	889	□	■	■	■	■	□	■	■	■	■

Note: CCB: coffee cascara from Brazil; CCN: coffee cascara from Nicaragua; K: kombucha; d0, d3, d6, and d9: days 0, 3, 6, and 9 of fermentation; ^a^ CAS# (Chemical Abstracts Service) Registry Number, available in the NIST database; ^b^ ELRI: Experimental Linear Retention Index; ^c^ LRI: Linear Retention Index based ontheliterature and NIST database [29]; 100% of the compounds in the chromatogram were identified. Alkanes were excluded. * Impact compounds according to: Ubeda et al. [71]; Pua et al. [84]; Nunes et al. [85]; Perestrelo et al. [88]; ^1^ http://www.flavornet.org (accessed on 7 June 2023) [75]; ^2^ http://www.thegoodscentsco.com (accessed on 7 June 2023) [76]. ■ compound identified in the sample. □ not identified.

Considering the volatile compounds in coffee cascara beverages according to chemical classes, nonanal, octanal, and dodecanal aldehydes were identified in all CCB and CCN beverages. Acetaldehyde was identified in all cascara kombuchas. This is a key product of fermentation and an inevitable component in wine [89]. Acetaldehyde has also been identified in a distilled, fermented coffee pulp beverage [90]. The number and area of alcohols increased from d3 to d9, especially phenylethyl alcohol and ethanol. Even if not identifiable on the olfactory level, ethanol is an important component of the kombucha aromatic profile [72]. Isoamyl alcohol (3-methyl-1-butanol) and 2-methyl-1-butanol, identified in CCB and CCN kombuchas, have also been identified in distilled, fermented coffee pulp beverages [90]. The contribution of acids to the global aroma depends on their concentration range. At low concentrations, acids with six to ten carbons provide a mild and pleasant aroma to wine [91]. The main acid responsible for kombucha sourness is acetic acid. Its concentration tends to increase with fermentation time [42,72]. Three volatile fatty acids were identified in CCB K and CCN K, decanoic acid, hexanoic acid (caproic acid), and octanoic acid. They have previously been identified during the production of sparkling wine, but only hexanoic and octanoic acids were mentioned as odor-active compounds [71].

Important esters identified during coffee cascara kombucha fermentation were ethyl acetate, isoamyl acetate, ethyl octanoate, ethyl hexanoate, ethyl decanoate, and ethyl isobutyrate. Most of them have also been identified in grape musts fermented by different yeasts [92]. Ethyl acetate was identified in a fermented coffee pulp distillate [90]. Ethyl decanoate and ethyl octanoate have been reported as abundant in ciders [77]. Ethyl isobutyrate, ethyl hexanoate, and isoamyl acetate have been reported as impact compounds in sparkling wine [71]. Regarding ketones, β-damascenone has been described as an aroma-active compound in a Robusta coffee pulp puree [93], while γ-nonalactone has been reported as the main odorant in red wine [88].

Monoterpenes were reported as volatile components of fruits responsible for a wide spectrum of aromas, mostly perceived as very pleasant [85]. Limonene was identified in CCB K d9 and CCN K d0, d3, and d6. Linalool was identified in all coffee cascara infusions and kombuchas. It has been previously identified in coffee cascara infusions [2].

### 3.4. Sensory Tests

#### 3.4.1. Rate All That Apply (RATA)

The RATA test was applied to characterize the sensory attributes of the kombucha samples and their intensities and relate them to the volatile composition presented in Section 3.3. A total of 113 consumers participated in the sensory assessment. The assessors’ characteristics are presented in Table 4.

**Figure 4:**
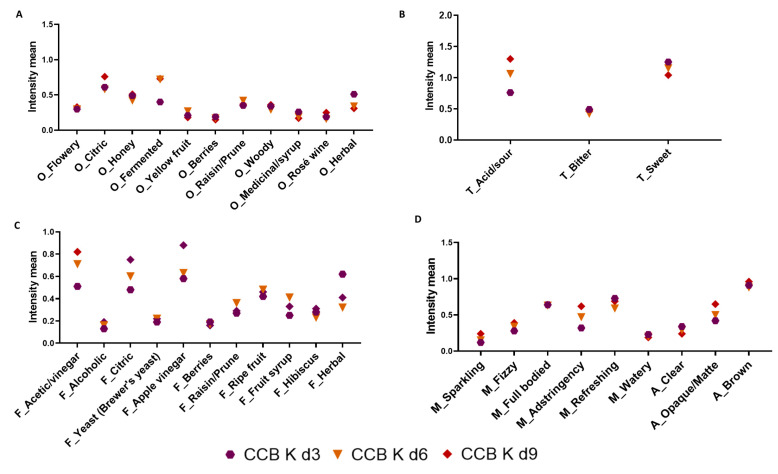
Intensity means for odor (**A**), taste (**B**), flavor (**C**), mouthfeel, and appearance (**D**) for CCB K d3, d6, and d9. Note: O: odor; T: taste; F: flavor; M: mouthfeel; A: appearance; CCB: Brazil coffee cascara.

**Figure 5:**
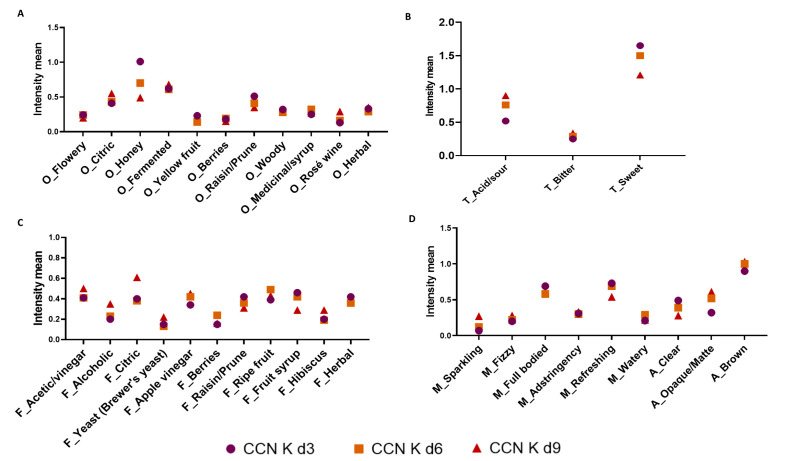
Intensity means for odor (**A**), taste (**B**), flavor (**C**), mouthfeel, and appearance (**D**) for CCN K d3, d6, and d9. Note: O: odor; T: taste; F: flavor; M: mouthfeel; A: appearance; CCN: Nicaragua coffee cascara.

Considering that kombucha is a fermented beverage, chemical changes generate different sensory attributes and intensities. Therefore, the RATA test was performed to identify these changes during the production of coffee cascara kombuchas. The intensity means for aroma, taste, flavor, mouthfeel, and appearance attributed to CCB Ks and CCN Ks by Rio de Janeiro consumers are presented in Figure 4 and Figure 5. Significant differences (*p* = 0.0001) in beverages made with the same raw material were observed mainly between 3d and 9d.

According to Kim and Adhikari [21] and Tran et al. [72], the main sensory characteristics of kombucha, such as sweet, sour, and vinegary odor and flavor, are developed via acetic acid bacteria activity and, cider odor and flavor and carbonation (from fermentation in general) via yeast activity. In addition to the important attributes that characterized CC K d3, attributes with significant intensity were observed by the assessors in CC K d9. They were citric, honey, fermented, and herbal for aroma; acid/sour and sweet for taste; alcoholic, apple vinegar, and herbal for flavor; slightly astringent and refreshing for mouthfeel; and opaque/matte and brown for appearance. A slight bitterness could be perceived in all kombuchas, which is attributed to non-volatile compounds such as caffeine and polyphenols in the raw materials [72]. No difference was perceived in bitterness intensity along the fermentation period.

Considering the two types of cascara, in general, CCB Ks obtained higher intensity means for flowery, citric, and fermented for odor; acid/sour for taste; acetic/vinegar, citric, apple vinegar, and hibiscus for flavor; and fizzy and astringent for mouthfeel. In CCB K d3, the attributes with higher intensities were berries, woody, and herbal for odor; sweet for taste; and herbal for flavor. On day 6, CCB K developed more sensory attributes, with high intensities of flowery, yellow fruit, and raisin/prune for aroma; acid/sour and bitter for taste; and acetic/vinegar, ripe fruit, and fruit syrup for flavor; and astringency appeared for mouthfeel. CCN Ks were described as fermented, woody, and rosé wine for odor; acid/sour and bitter for taste; and alcoholic and citric for flavor. In CCN K d3, the attributes showing higher intensities were honey, berries, and raisin/prune for odor; sweet for taste; raisin/prune and fruit syrup for flavor; and full-bodied for mouthfeel. The sensory complexity attributed to CCN K d3 is most probably derived from the fact that CCN was obtained from previous fruit fermentation during the post-harvest process [2]. For CCN K d6, assessors marked higher intensity for berries and medicinal/syrup for aroma; sweet for taste; citric, berries, and ripe fruit for flavor; and watery for mouthfeel. However, as previously stated, this sample received a lower acceptance score than CCB K d3, probably because of the increase in acetic/vinegar flavor together with the positive attributes (Figure 3).

Reports on some spirits prepared with coffee cascara were found in the literature. Einfalt et al. [94] produced fresh coffee cherry spirits, with vegetable, nutty, and earthy aroma attributes, while the taste descriptors were vegetable, alcoholic, and nutty. Blumenthal et al. [90] also produced cherry spirits from different arabica coffee varieties and found different sensory results and preferences among the coffee plant varieties. In both studies, woody, plum, compote/jam, sweet, herbs, dried fruits, stone fruit, and cherry-like were cited attributes. These descriptors are in accordance with the present study results, partly explaining the differences between CCB and CCN during K production.

Higher intensity means for acid/sour are usually attributed to the organic acid concentration reflected in higher TA and lower pH values [95], which was also observed in the present study. It is known that sourness decreases the ability of humans to detect the initial sweetness of sucrose, given that the sucrose threshold stabilizes with the increase of acid in the same medium [96]. This can help explain the low intensity mean for sweet taste in kombuchas fermented for 9 days. In CCN K, some of the attributes with high intensities used to describe d9 samples were brewer’s yeast for flavor, sparkling for mouthfeel, and more opaque/matte for appearance. In CCB K, some of these attributes presented similar intensities in all samples.

**Figure 6:**
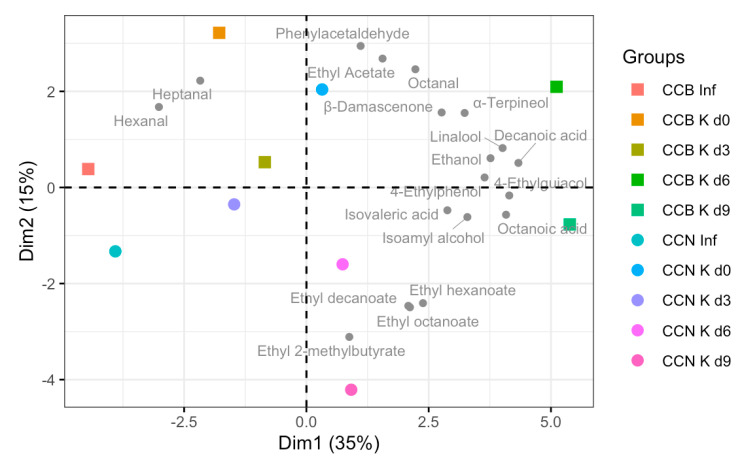
Biplot of Principal Component Analysis (PCA) using as variables the volatile compounds identified as relevant for RATA attributes summarized in Table 5. CCB: coffee cascara from Brazil; CCN: coffee cascara from Nicaragua; Inf: infusion; K: kombucha; d0, d3, d6, and d9: days of fermentation.

Table 5 contains the RATA attributes that, based on data from the literature, could correspond to the volatile compounds identified in this study. Principal Component Analysis was performed using as variables the volatile compounds summarized in Table 5; the biplot obtained (Figure 6) highlights that, in general, the evolution of the profile of those selected volatiles during fermentation is in agreement with the changes observed in sensory characteristics. Similar changes for the profile of selected volatiles of CCB and CCN from infusion to d3 were observed, while at d6 and d9 relevant differences were noted between CCB K and CCN K, which were all observed in the RATA test.

#### 3.4.2. Consumer Acceptance and Purchase Intent Test Scores

**Figure 7:**
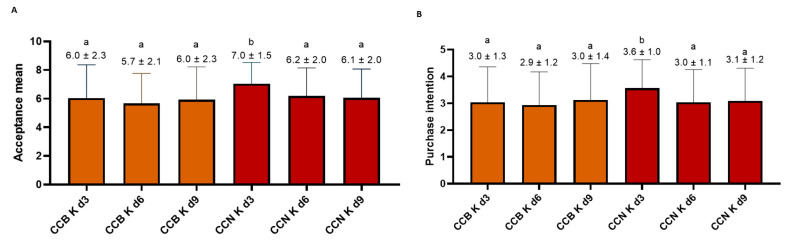
Mean acceptance (**A**) and purchase intention (**B**) scores of CC Ks by Rio de Janeiro consumers *n* = 113). Note: CCB K: coffee cascara from Brazil; CCN K: kombucha made with coffee cascara from Nicaragua; d3: day 3 of fermentation; d6: day 6; d9: day 9. Different letters over the bars indicate significant statistical difference between them.

Because of the variability in acceptance scores, the mean acceptance (6.0 ± 0.14) was similar for all CCB K, with no statistical difference. Similar results were obtained for CCN K, except for sample 3d, which scored 7.0, with 89% of the scores between 6 and 9 (Figure 7A); therefore, all samples were accepted. Purchase intent results followed the same trend (Figure 7B).

According to Meilgaard et al. [32], for a sample to be considered “well-accepted”, it must obtain an Acceptance Index (AI) equal to or higher than 70%. CCN d3 reached 78% AI, while other samples had AI between 63% and 69%. Therefore, CCN K d3 was the only generally well-accepted kombucha. Such higher acceptance is probably derived from the higher amount of sugar, highlighted by the lower acidity and volatile compounds with sweet, floral, and fruity notes in the infusion used for kombucha preparation, given that these are suitable attributes for kombucha in general [95]. Brazilians in general are used to sweeter beverages, a habit inherited from the Portuguese colonizers who are now educated by the European public health agencies to lower sugar consumption, just like other European countries [117]. The United States (US) follows the low sugar trend [118]. As mentioned previously, the amount of sugar contained in the kombucha market in the US and Europe can be considerably low, which makes this beverage an excellent replacement for soft drinks and other nutritionally poor beverages, usually containing more than 10 g sugar/100 mL. It is worth mentioning that in preliminary tests, the fermentation period necessary to achieve the desired sensory characteristics was related to the proportion of starter and other ingredients, type and variety of raw materials, size of the bottle, volume of kombucha, and so forth. Longer fermentation with a lower initial amount of sugar and the addition of low glycemic index sweeteners will probably reach the same desirable sensory result as the product developed in this study.

Because people have different tastes and experiences and cannot be represented by a mean score, we performed a cluster analysis to identify different niches of consumers. Two clusters were identified:

Cluster 1 (*n* = 45, mean score = 7.3, and AI = 81%) consistently attributed the highest scores to CCB K d9 (mostly male, acceptance mean = 7.8) and CCN K d3 (mostly female, acceptance mean = 7.5). This cluster was composed of 52% male and 48% female. Forty-two percent of them were 18–24 years old. Forty-nine percent had higher education, and 38% had a family monthly income of 2–3 MW. In this cluster, 8% of the assessors were kombucha consumers. The women’s attribution of higher scores to CCN K d3 may be related to the fact they regularly drank sweetened (36%) teas with fruity notes, consumed other sweet beverages such as soft drinks (36%), and reported low consumption of sparkling water (17%), sparkling wine/cider (15%), and tonic water (15%). DePaula et al. [2] also have recently observed that women aged 18–34 habitually consume fruity teas, confirming data from the Brazilian Institute of Statistics and Geography (IBGE) [119]. Because our assessors were mostly women (61%), this could be the main reason why CCN K d3 received the highest average score. The men’s attribution of higher scores to CCB K d9 can be explained by the high consumption of different sparkling and fermented beverages. This sample was not only more acidic, but it also had a high intensity mean for bitter taste and sparkling mouthfeel. Beer, sparkling water, and tonic water are bitter and sparkling beverages. Thirty-seven percent of men reported consumption of sparkling water, 22% tonic water, and 30% sparkling wine and/or cider. According to IBGE [119], young Brazilian men drink more soda, while adult men drink more soda and beer than women.

Cluster 2 (*n* = 67, mean score = 5.3, and AI = 59%) also attributed the highest scores to CCN K d3. This cluster was also primarily composed of females (72%) but aged between 25 and 34 (58%), with complete graduate education (54%) and family monthly income between 2 and 3 MW (53%). Nine percent of assessors were kombucha consumers. Similar characteristics to cluster 1 related to gender were observed.

Together, these findings suggest that young adults are potential consumers of cascara kombuchas, although assessors were mostly young because only they were willing to participate in the study (Table 4). The results also indicate that there are potential market niches for kombuchas with different intensities of fermentation, including lower levels of fermentation for women and higher levels for men, although in North America and Europe, higher intensities of fermentation would probably receive higher scores in general, considering the existing products on their market shelves.

No study on coffee cascara kombucha was found for comparison, but considering the sensory acceptance of kombuchas made with other new substrates (black carrot, cherry laurel, blackthorn, and red raspberry) in the study by Ulusoy and Tamer [40] performed in Turkey, the beverages fermented for shorter periods (3 and 5 days) obtained scores between 6 and 8, using a 9-point hedonic scale, while beverages fermented for 10 and 12 days received scores below 5. Other studies performed in Brazil and Tunisia reported that assessors liked herbal and grape kombuchas after 6 days of fermentation, with average acceptance scores between 5 and 7 [49,120]. Unfortunately, a comparison regarding gender and age was not possible because such information was not available in these studies.

## 4. Conclusions and Final Considerations

In the present study, 81 volatile organic compounds were identified considering infusions and fermented beverages. Amounts of 24 and 28 compounds were identified in CCB and CCN infusions, respectively, and 22 in BT infusions. The volatile profile changed dramatically during fermentation, with 59 compounds commonly identified in all kombuchas. Despite different origins and post-harvest processing, both groups of coffee cascara kombucha presented similar volatile profiles. The content of acids and esters increased progressively due to the symbiosis between acetic acid bacteria and yeasts, represented in the consortia used in this study mostly by the genera *Komagateibacter* sp. and *Pichia* sp., respectively.

Coffee cascara kombucha was accepted by the assessors from Rio de Janeiro in general, especially the one containing a higher amount of sugar and fruity and flowery attributes, resembling the Guaraná soft drink commonly consumed by Rio de Janeiro’s population. Young adults showed to be potential consumers of coffee cascara kombucha, with women preferring the early stages of fermentation and men later stages. The sensory characterization was associated with the volatile composition of the beverages. Volatile compounds that seem to have contributed the most to the main characteristics of coffee cascara kombucha were linalool, decanal, nonanal, octanal, dodecanal, ethanol, 2-ethylhexanol, ethyl acetate, ethyl butyrate, β-damascenone, γ-nonalactone, ethanol, linalool oxide, phenylethyl alcohol, phenylacetaldehyde, isoamyl alcohol, acetic acid, octanoic acid, decanoic acid, ethyl isobutyrate, ethyl hexanoate, and limonene. However, this hypothesis needs to be confirmed by studies involving gas chromatography analysis with olfactometric detection.

Coffee cascara showed to be a suitable raw material to produce aromatic and natural cold beverages, that with reduced amounts of sugar and caffeine (compared to soft drinks and other stimulant beverages) [20,121], and a considerable number of bioactive compounds, can be an excellent replacement for nutritionally poor soft drinks and a way to reduce environmental pollution caused by the incorrect disposal of coffee cascara after harvest, as well as improving the coffee chain sustainability. Additionally, different from most kombuchas which are flavored to increase acceptance, coffee cascara kombuchas do not need flavoring agents like fruits, herbs, or spices commonly used for the traditional kombucha or kombucha-like beverages. Therefore, the potential of coffee cascara kombuchas to produce healthy fermented beverages like kombucha is remarkable.

## Figures and Tables

**Table 1:** Physicochemical characterization and sucrose content in black tea and coffee cascara kombuchas.

	Days of Fermentation	Titrable Acidity (mEq/L)	pH	Soluble Solids (°Brix)	Sucrose (g/100 mL)
Black tea	0	0.1 ± 0.00 ^a^	3.8 ± 0.07 ^a^	10.4 ± 0.07 ^a^	10.5 ± 0.75 ^a^
	3	0.2 ± 0.05 ^a^	3.5 ± 0.00 ^b^	10.8 ± 0.14 ^a^	9.1 ± 0.75 ^b^
	6	0.3 ± 0.06 ^b^	3.5 ± 0.00 ^b^	9.7 ± 0.00 ^b^	8.2 ± 0.75 ^c^
	9	0.3 ± 0.06 ^b^	3.4 ± 0.00 ^b^	9.3 ± 0.28 ^b^	7.9 ± 0.75 ^d^
CCB K	0	0.1 ± 0.00 ^a^	3.7 ± 0.07 ^a^	11.5 ± 0.63 ^a^	10.6 ± 0.26 ^a^
	3	0.2 ± 0.00 ^a^	3.6 ± 0.07 ^a^	11.3 ± 0.00 ^a^	10.5 ± 0.25 ^a^
	6	0.2 ± 0.00 ^a^	3.6 ± 0.07 ^a^	10.4 ± 0.14 ^b^	9.4 ± 0.75 ^b^
	9	0.2 ± 0.00 ^a^	3.5 ± 0.00 ^a^	10.0 ± 0.42 ^b^	8.2 ± 0.75 ^c^
CCN K	0	0.04 ± 0.00 ^a^	3.8 ± 0.21 ^a^	11.6 ± 0.14 ^a^	10.5 ± 0.75 ^a^
	3	0.2 ± 0.00 ^b^	3.6 ± 0.07 ^a^	10.9 ± 0.14 ^a^	9.0 ± 0.75 ^b^
	6	0.2 ± 0.05 ^b^	3.5 ± 0.00 ^a^	10.6 ± 0.49 ^a^	8.5 ± 0.75 ^c^
	9	0.4 ± 0.08 ^b^	3.5 ± 0.00 ^a^	9.9 ± 0.56 ^b^	8.1 ± 0.75 ^d^

Data are expressed as mean ± standard deviation for triplicate analyses; different letters on the same column for the same beverage indicate significant difference (*p* < 0.05); CCB K: kombucha made with coffee cascara from Brazil; CCN K: kombucha made with coffee cascara from Nicaragua.

**Table 4:** RATA assessors’ characteristics.

Gender		Age			
Male	Female	18–24	25–34	34–44	45–59
39%	61%	47%	38%	6%	8%
Level of Education					
Basic education	Undergraduate	Incompletegraduation	Completegraduation	Master’s ordoctoral degree	
0%	12%	40%	10%	38%	
Family Income (MW: Minimum Wages)					
1 MW	2–3 MW	4–5 MW	>5 MW		
18%	39%	9%	7%		
Know Kombucha		Drink Kombucha			
Yes	No	Yes	No		
68%	32%	9%	81%		
Sparkling Beverage/Soft Drink Consumption					
Sparkling water	Apple juice	Soda	Tonic water	Sparkling wine	Cider
50%	10%	73%	32%	42%	15%

**Table 5:** RATA attributes and the corresponding volatile compounds identified in the present study for coffee cascara kombuchas (see Table 3).

Aroma and Flavor Attributesfrom RATA Test	Respective Volatile Compounds Identified in the Literature and in the Present Study	References
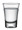 Alcoholic	Isoamyl alcohol, ethyl acetate, ethyl hexanoate, ethyl decanoate, linalool oxide	[97]
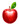 Apple	Dodecanal, heptanal benzaldehyde, decanal, decanoic acid, ethyl 2-methylbutyrate, ethyl butyrate, methyl salicylate	[98]
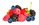 Berries	Hexanal, decanal, benzaldehyde, octanoic acid, ethyl acetate, ethyl hexanoate, ethyl octanoate, linalool	[98,99,100]
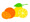 Citric	Nonanal, octanal, dodecanal, limonene α-terpineol, linalool	[30]
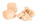 Fermented	Hexanal, benzaldehyde, nonanal, phenylacetaldehyde, ethanol, octanoic acid	[101]
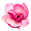 Floral/flowery	Phenylethyl alcohol, β-damascenone, linalool, linalool oxide, cis/trans-linalool oxide	[102]
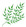 Herbal	Hexanal, heptanal, nonanal, octanal, phenylacetaldehyde, phenylethyl alcohol, linalool	[103]
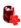 Hibiscus	Dodecanal, nonanal, 2-ethylhexanol, acetic acid, linalool	[104]
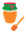 Honey	Octanal, benzaldehyde, phenylacetaldehyde, phenylethyl alcohol, isovaleric acid, β-damascenone, cis/trans-linalool oxide, linalool, α-terpineol	[105,106]
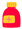 Medicinal syrup	4-ethylguiacol, 4-ethylphenol	[107]
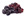 Raisin/prune	Nonanal, benzaldehyde, ethyl octanoate β-damascenone, γ-nonalactone, linalool	[85,86]
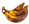 Ripe fruit	Hexanal, hexanoic acid, octanoic acid, ethanol, ethyl acetate, isoamyl acetate, cis/trans linalool-oxide, linalool	[108,109]
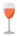 Rosé wine	Nonanal, 1-dodecanol, 1-heptanol, octanoic acid, decanoic acid, ethyl hexanoate, ethyl octanoate, ethyl decanoate, β-damascenone, linalool	[110]
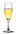 Sparkling wine	Hexanoic acid, octanoic acid, ethyl isobutyrate, ethyl hexanoate, isoamyl acetate	[71]
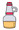 Vinegar	Acetic acid	[72]
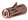 Woody	Hexanal, nonanal, cedrol, acetic acid, decanoic acid	[111,112]
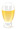 Brewer’s yeast	Nonanal, ethanol, octanoic acid, isoamyl alcohol, ethyl decanoate, ethyl hexanoate	[113]
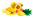 Yellow fruit	Benzaldehyde, heptanal, nonanal, octanal, ethyl butyrate, ethyl octanoate, ethyl acetate, β-damascenone, limonene, α-terpineol	[114,115,116]

## Data Availability

The data presented in this study are available on request from the corresponding author.

## Supplementary Materials

The following supporting information can be downloaded at: https://www.mdpi.com/article/10.3390/foods12142710/s1: Figure S1. Total Ion Chromatograms (TIC) of kombuchas obtained by SPME/GC/MS; Figure S2. Calibration curves of sucrose (A), glucose (B), and fructose (C) standards used for sugar analysis via High Performance Liquid Chromatography Refractive Index Detector system.
