# Supplementary material for: Volatile, Microbial, and Sensory Profiles and Consumer Acceptance of Coffee Cascara Kombuchas
Source: Foods. 2023 Jul 15;12(14):2710. doi: 10.3390/foods12142710 (PMC10379779; doi:10.3390/foods12142710)
Supplement: Supplementary file 1 [file foods-12-02710-s001.zip › foods-2475266-supplementary.pdf]

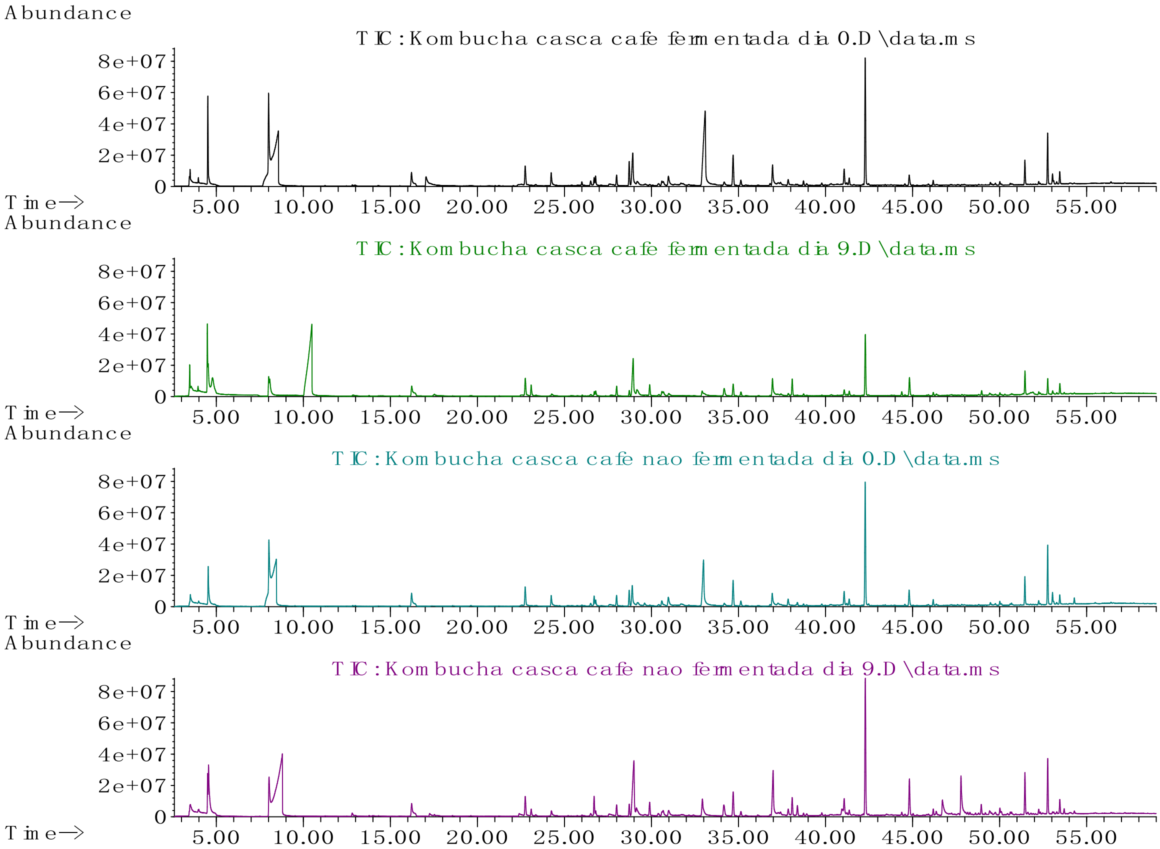

Figure S1. Total Ion Chromatograms (TIC) of kombuchas obtained by SPME/GC/MS

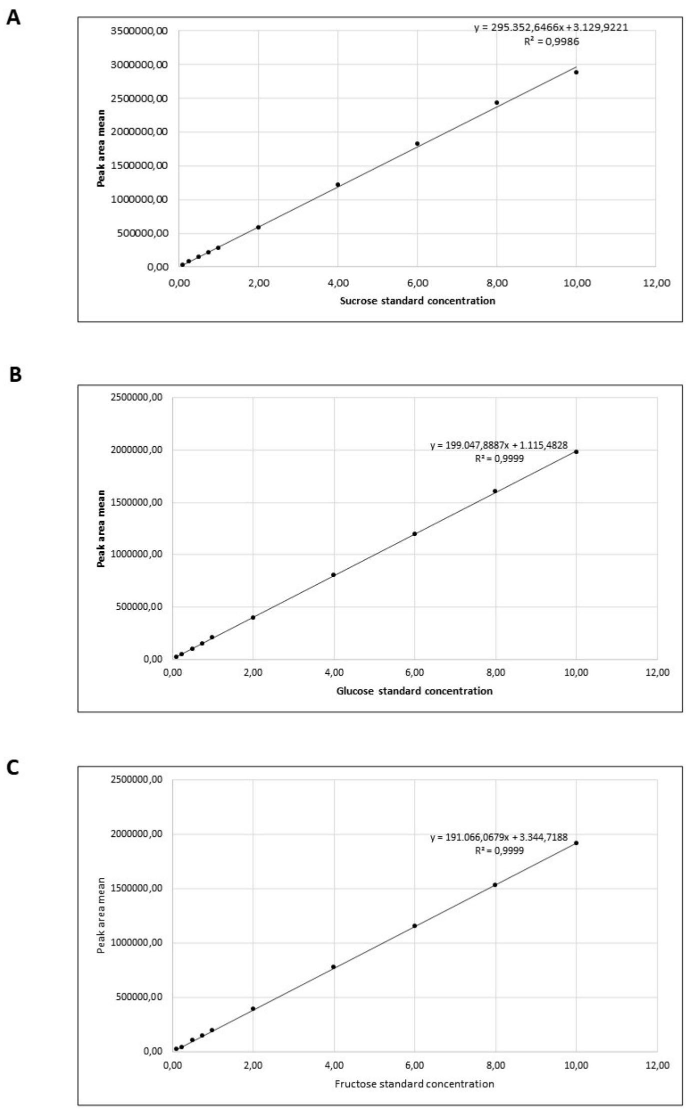

Figure S2. Calibration curves of sucrose (A), glucose (B) and fructose (C) standards used for sugars analysis by High Performance Liquid Chromarography Refractive Index Detector system.
